# Efficient clinical decision-making process via AI-based multimodal data fusion: A COVID-19 case study

**DOI:** 10.1016/j.heliyon.2024.e38642

**Published:** 2024-10-10

**Authors:** Daniel I. Morís, Joaquim de Moura, Pedro J. Marcos, Enrique Míguez Rey, Jorge Novo, Marcos Ortega

**Affiliations:** a Varpa Group, Biomedical Research Institute A Coruña (INIBIC), University of A Coruña, 15006, A Coruña, Spain; b Department of Computer Science and Information Technologies, University of A Coruña, 15071, A Coruña, Spain; c Dirección Asistencial y Servicio de Neumología, Complejo Hospitalario Universitario de A Coruña (CHUAC), Instituto de Investigación Biomédica de A Coruña (INIBIC), Universidade da Coruña, Sergas, 15006 A Coruña, Spain; d Grupo de Investigación en Virología Clínica, Sección de Enfermedades Infecciosas, Servicio de Medicina Interna, Instituto de Investigación Biomédica de A Coruña (INIBIC), Área Sanitaria A Coruña y CEE (ASCC), SERGAS, 15006 A Coruña, Spain

**Keywords:** Information fusion, Risk estimation, Clinical data, Deep features, COVID-19, Chest X-ray

## Abstract

COVID-19 is an infectious disease that caused a global pandemic in 2020. In the critical moments of this healthcare emergencies, the medical staff needs to take important decisions in a context of limited resources that must be carefully managed. To this end, the computer-aided diagnosis methods are extremely powerful and help them to better recognize the evidences of high-risk patients. This can be done with the support of relevant information extracted from electronic health records, lab tests and imaging studies. In this work, we present a novel fully-automatic efficient method to help the clinical decision-making process in the context of COVID-19 risk estimation, using multimodal data fusion of clinical features and deep features extracted from chest X-ray images. The risk estimation is studied in two of the most relevant and critical encountered scenarios: the risk of hospitalization and mortality. This study shows which are the most important features for each scenario, the ratio of clinical and imaging features present in the top ranking and the performance of the used machine learning models. The results demonstrate a great performance by the classifiers, estimating the risk of hospitalization with an AUC-ROC of 0.8452 ± 0.0133 and the risk of death with an AUC-ROC of 0.8285 ± 0.0210, only using a subset of the original features, and highlight the significant contribution of imaging features to hospitalization risk assessment, while clinical features become more crucial for mortality risk evaluation. Furthermore, multimodal data fusion can outperform the approaches that use one data source. Despite the model's complexity, it requires fewer features, an advantage in scenarios with limited computational resources. This streamlined, fully-automated method shows promising potential to improve the clinical decision-making process and better manage medical resources, not only in the context of COVID-19, but also in other clinical scenarios.

## 1 Introduction

The COVID-19 is a challenging lung disease that caused the emergence of a health crisis in 2020, as it was declared as global pandemic in that year by the World Health Organization (WHO) [55]. By the time this manuscript was written, more than 767 million cases were confirmed, including a total of more than 6.9 million deaths [61]. The great impact of the COVID-19 in the healthcare systems meant a challenge that led to significant rates of hospitalization and ICU admission, specially during the first phases of the pandemic, when the evolution of the disease was still uncertain. When such situations occur, it is critical to manage the resources more effectively, providing a greater attention to the high-risk patients.

When someone that is suspected of having COVID-19 reaches an Emergency Room or is admitted to the hospital, the clinicians must find their Electronic Health Records (EHR) that provide relevant health information about the patients. This health information includes demographic data and other relevant variables as the preconditions or the treatments of the patient, and can help the clinicians to identify high-risk individuals [43]. These data are usually complemented with additional testing. This is the case of the laboratory blood tests, that provide a picture of the patient health state in a particular moment [31]. An insight of the patient state in a given moment can also be provided by imaging tests [42]. In particular, given that the COVID-19 mainly affects to the lungs, the most used imaging tests are the ones that visualize this area and surrounding tissues. This visualization can be done with chest X-ray imaging [48] and with more advanced radiological capturing methods, such is the case of Computed Tomography (CT) [44]. However, in a critical situation of health emergency, the CT imaging modality is inappropriate. The CT imaging is more invasive (as it needs a greater amount of X-ray to be performed), more expensive and takes a greater time to perform the captures, some aspects that are undesirable in an emergency context. Against this, the chest X-ray devices offer some great advantages. Particularly, chest X-ray captures are much cheaper and easier to perform and can be obtained quicker than a CT capture. Moreover, many patients suffer from a severe form of COVID-19, leaving them bedridden or with difficulties to be displaced to a radiology room. To solve this problematic, the health emergency rooms consider the use of portable chest X-ray devices, that can be moved to where the patient is placed. It is worth mentioning an additional advantage of chest X-ray, to tackle the problem of cross-contamination, given that the greater complexity of CT machinery and the greater time of contact between the patient and the device, makes the process of decontamination more difficult. However, all these advantages are accompanied by a lower quality and level of detail in comparison with the more advanced imaging techniques.

To help clinicians in their decision-making processes, the Computer-Aided Diagnosis (CAD) methods have shown to be a very powerful tool in the last decades [14]. This kind of tools allows analyzing the most relevant features to estimate the risk of a patient. The analysis can be done using EHR, determining the most relevant demographics, preconditions, and treatments that are more likely to worsen the outcome of a COVID-19 patient. Nevertheless, the same study can be performed using other different sources of data, like the mentioned chest X-ray images, given that the found evidences could be decisive to determine the patient risk. Furthermore, as clinicians usually make decisions evaluating different data sources in their daily practice, the same approach could be extrapolated to the scope of CAD methods. This means that clinical features could be complemented with imaging features to improve the performance of the overall system. It is also worth to mention the current interest on explainable artificial intelligence (XAI) methodologies, that has significantly influenced the works of the state-of-the-art. This interest is motivated by the fact that XAI methodologies can improve the performance of existing methods [6], address ethical and legal issues [18], understand the findings of the models and discuss if they match with those provided by the clinical literature [34] and to make it easier for clinicians to accept and trust the implemented technology [28].

Due to the significant impact that COVID-19 has had worldwide, many contributions of Computer-Aided Diagnosis (CAD) methods have been proposed in this field, with different types of data sources. For the purposes of our study, 3 specific types of works must be mentioned: those that use chest X-ray imaging, those that use clinical data and those that fuse these 2 different data sources.

• **Chest X-ray imaging:** many works of the state-of-the-art have leveraged deep learning methods to detect the COVID-19 in chest X-ray images [3]. These contributions use heterogeneous datasets, with a wide range of different sizes, class imbalances, capture devices and building criteria (regarding the number of classes, among other aspects). In particular, some proposed approaches present an end-to-end methodology based on deep network architectures. In contrast with that, other works propose pipelines that include a process of feature extraction followed by a classification, that leverages the set of extracted features. This feature extraction is performed using deep network architectures, obtaining a set of deep features, but also with other classical strategies such as radiomics [41] or shape and texture descriptors. The aim of these works is to find evidences on images that can determine if a patient has COVID-19 or not, or if the evidences present a potential scenario of a more or less severe form of the disease within a time frame. To complement these studies, some contributions also include a final phase of explanation, with algorithms like GradCAM [45] or Gradient Backpropagation [7], among others.
• **Clinical data:** the potential of using EHR in conjunction with machine learning has been demonstrated to predict patient outcomes in scenarios like heart failure, postoperative evolution, as well as other cardiovascular and pulmonary risk scenarios [49]. This potential has also been considered in the field of COVID-19 to identify health risks, monitor possible complications and support the clinical decisions [43].
• **Multimodal data fusion:** despite the proposal of machine learning methodologies applied to a single modality are interesting, the potential of leveraging multiple available data sources to obtain a better global performance is left unexploited. In particular, some works of the state-of-the-art combine multimodal datasets from data sources that can consist of omics, laboratory tests, imaging and physiological characteristics, among others [11]. Within this research line, there are scenarios that combine features extracted from sources of chest X-ray images and clinical data obtained from EHR. This can be performed either by extracting features from images and then concatenating them to the clinical data, or by implementing an end-to-end approach with a deep network architecture capable of processing both inputs simultaneously.

While significant progress has been made in utilizing multimodal data fusion for COVID-19 research, substantial limitations persist, marking key areas for further exploration. These limitations include the under-explored comprehensive analysis of key features derived from the fusion of clinical and imaging data and the neglected examination of how each data source influences risk estimation models' performance. Additionally, the process of feature selection demands a more efficient approach. Efficient feature selection not only minimizes the input dataset size but also mitigates computational memory requirements, which is especially crucial in clinical environments where resources may be limited. To bridge these gaps, our study presents an innovative methodology that harnesses the power of multimodal data fusion and machine learning to accurately estimate COVID-19 risk. This work transcends traditional methods, introducing an efficient, fully-automated decision-making tool that is highly applicable to clinical settings grappling with COVID-19 and potentially other medical scenarios. Key highlights of our study are:

• The introduction of a fully-automatic, efficient machine learning-based method aimed at enhancing decision-making processes in clinical settings for COVID-19.
• The implementation of multimodal data fusion that seamlessly integrates clinical data from electronic health records with feature information extracted from chest X-ray images.
• The extraction of imaging features from three distinct layers of a well-established deep network architecture, thus evaluating different levels of feature granularity.
• The utilization of an efficient feature selection process that identifies and prioritizes the most critical features, a pivotal factor in resource-constrained medical environments.
• The training of a widely-used machine learning model using these critical features to estimate COVID-19 patient risk across two relevant scenarios: risk of hospitalization and risk of death.
• The evaluation of the multimodal approach by the means of a comprehensive series of experiments that compared the effectiveness of relying solely on clinical data, solely on imaging data and integrating both modalities.

The rest of the manuscript is structured as follows. Firstly, Section 2 discusses the main contributions that can be found related with our work. After that, the used dataset, software and hardware are described in Section 3. Then, the methodological proposal is explained in Section 4, followed by the report of the results, with their corresponding discussion in Section 5. Finally, the main conclusions and possible lines of future works are detailed in Section 6.

## 2 Related works

Given the great relevance that the COVID-19 has had worldwide during the last years, the research community has proposed a vast amount of contributions, to solve several tasks with different data modalities. In this section, we discuss the main works closely related to our proposal that can be found. This section has been divided in different subsections, one for each data source. Particularly, the Subsection 2.1 discusses the previous state-of-the-art works that use chest X-ray imaging in the context of COVID-19. Afterward, the Subsection 2.2 addresses the study of the works that train with only clinical data. Finally, the Subsection 2.3 describes the work related with multimodal data fusion.

### 2.1 Chest X-ray imaging

Many works have proposed methodologies to perform tasks to support clinicians using chest X-ray images in the context of COVID-19. As reference, the work from [37] performs a prediction of the COVID-19 patients that are in a risk of death using radiomics extracted from chest X-ray images, that are finally fed to a machine learning model that can be a Linear Discriminant Analysis model or a Support Vector Machine. Shankar et al. [46] develops a method for COVID-19 diagnosis based on the extraction of classical texture descriptors from chest X-ray images (in particular, the Local Binary Patterns, the Gray Level Co-occurrence Matrix and the Gray Level Run Length Matrix). Once these texture descriptors' features are fused, the most relevant are selected. Finally, this set of features is fed to a Convolutional Neural Network (CNN) that makes the classification process. De Moura et al. [13] presents a methodology to analyze chest X-ray images to distinguish between healthy patients, patients with pneumonia affectation different from COVID-19 and COVID-19 patients using 6 different deep network architectures (2 DenseNet, 2 ResNet and 2 VGG-16) and public datasets. In another contribution by De Moura et al. [12], the authors propose a fully automatic method to classify portable chest X-ray images in 3 different categories (normal, pathological and COVID-19) adapting the architecture of a DenseNet-161. Vidal et al. [58] proposed a pulmonary-restricted methodology designed to extract features exclusively from the pulmonary region of interest. They employed a sophisticated approach, utilizing multi-stage transfer learning for lung segmentation [57], making their technique particularly beneficial when analyzing intricate portable images. Their methodology goes beyond mere feature extraction; it also generates a class activation map, thereby adding a layer of explainability to the model's results. In the case of [27], the authors propose a deep architecture for COVID-19 classification that fuses the features extracted from 5 models: EfficientNet-B0, MobileNet-V2, Inception-V3, ResNet-50 and ResNet-101. Then, this set of fused features is fed to a Support Vector Machine. Another interesting contribution is the one provided by Ho & Gwak [19], that fuses radiomics with classical handcrafted features and deep features obtained from a pretrained ResNet-18 and a DenseNet-121 (all of them extracted from chest X-ray images) to detect the COVID-19, using classical machine learning classifiers. The work from [20] uses radiomics to complement 3 CNN architectures (VGG-16, VGG-19 and DenseNet-121) and improve their performance to discriminate between COVID-19 and NON-COVID-19 pneumonia in chest X-ray images.

### 2.2 Clinical data

Some studies focus exclusively on the utilization of clinical data for diagnosing and assessing COVID-19 patient risk. As reference, the work of Laatifi et al. [26] proposes the application of machine learning algorithms to evaluate the influence of cytokines on the severity of a COVID-19 infection, adding explainability with the Shapley additive explanation (SHAP) algorithm and Local Interpretable Model-agnostic Explanations (LIME). On their hand, in [40], the authors propose a methodology to predict the severity of COVID-19 patients at hospital admission, measured with the risk of needing mechanical ventilation and 30-day mortality. In the case of [60], the authors provide a method to predict COVID-19 mortality risk using machine learning models, reporting the performance when applying the methodology over data sources from different health centers. Liu et al. [30] analyze the performance of using machine learning to diagnose the disease and predict its severity using different sources of omic-data. Other interesting contribution is the one from [36], that uses different machine learning models (Support Vector Machine, Decision Tree, XGBoost, Multilayer Perceptron and k-Nearest Neighbors) to estimate the outcome of COVID-19 patients in 2 different scenarios: estimate the risk of hospitalization and the risk of death. Another representative work in this scope is the contribution of Emami et al. [16], where the authors propose the application of 4 different machine learning models (Support Vector Machine, Gradient Boosting Tree, Random Forest and Regression Logistic) to predict COVID-19 mortality, using different types of tabular data: demographics, history of the patients' risk factors, laboratory test results and other potentially relevant information. Finally, it is remarkable the work of Polilli et al. [38], that proposes a methodology to predict the risk of hospitalization, necessity of oxygen support, intensive therapy and death using Logistic Regression and Cox modeling.

### 2.3 Multimodal data fusion (chest X-ray with clinical data)

The integration of different data sources has also been explored in the context of COVID-19, something that can be found with the particular case of fusing clinical data with chest X-ray imaging. As reference, the work of Wu et al. [62] fuses the information provided by chest X-ray images of the patients with their EHR to predict 30-day COVID-19 mortality. In particular, a deep model is trained to predict this risk with only the chest X-ray capture and, then, this prediction is merged with the set of features. The fusion of these characteristics is then fed to the classifiers. For this aim, the approach uses 4 different machine learning models: XGBoost, Gradient Boost, Logistic Regression and Random Forest. In the case of [25], the authors propose a deep network architecture with the ability to concatenate clinical data and imaging data (represented by the chest X-ray captures of the patients) to perform a straightforward end-to-end COVID-19 diagnosis. This approach is compared against training only with clinical data and only with imaging data, but following the same end-to-end philosophy. In the case of [63], the authors proposed the architecture DeepCOVID-Fuse, that fuses the features of chest X-ray images extracted from 3 deep network architectures (EfficientNet-B2, ResNet-50 and DenseNet-121) and clinical features to predict 3 levels of risk: low, intermediate and high. The low-risk category indicates hospital stay of less than one day, the intermediate-risk indicates hospital stay of more than one day without death or ICU admission and high-risk indicates death or ICU admission. The architectural proposal allows obtaining the outcome directly from the input data, in an end-to-end manner.

Another interesting work that can be included in this literature exploration is [52]. This contribution proposes three approaches of clinical outcomes in COVID-19 patients fusing clinical data and chest X-ray images. The first approach contemplates the fusion of clinical data with chest X-ray features that were extracted by the means of handcrafted strategies and then inputted to three classical machine learning algorithms (Support Vector Machine, Random Forest and Logistic Regression). The second approach contemplates the same pipeline as in the first case, but replacing the handcrafted imaging feature extraction by a deep feature extraction. Finally, in the third approach, the pipeline is replaced by an end-to-end scheme, that inputs both the clinical data and imaging data without requiring any additional feature extraction steps. The work of Wang et al. [59] uses CT images of COVID-19 patients to predict the disease progression, classifying each patient in two categories: aggravation or improvement. This pipeline includes the extraction of radiomic features from CT images, to train the so-called radiomics model, a classical machine learning classifier that can be Logistic Regression, Support Vector Machine, Decision Tree, Random Forest or XGBoost. Then, another classifier is trained with demographic data and laboratory test results (the so-called clinical model). Furthermore, the authors of the work also contemplate the exploration of fusing both data sources to train the so-called combined model. Finally, it is also worth to mention the work of Prinzi et al. [39] that extracts radiomic features from chest X-ray images and combines them with clinical and laboratory data to train a Support Vector Machine and a Random Forest model, considering three feature selection algorithms and including explainability mechanisms, with the aim to perform a COVID-19 prognosis prediction.

## 3 Materials

In this section, we present the materials that are required for the development of this work. In particular, Subsection 3.1 presents the characteristics of the dataset and Subsection 3.2, the software and the hardware that were used to perform the experimentation. This section provides the necessary information to replicate the proposed methodology.

### 3.1 Dataset

The dataset used in this work was supplied by the Complexo Hospitalario Universitario de A Coruña (CHUAC), Galicia, Spain and specifically retrieved for the purposes of this work. The data is composed of 2,040 patients that were confirmed as COVID-19 positives. These patients are modeled as a set of 28 clinical features, including a subset of demographic variables (Age and Age Range [5], the Sex [4], Height, Weight and Body Mass Index, abbreviated as BMI [1]), a second subset of health preconditions and treatments with a potential relevance to determine the outcome of the COVID-19 patients, a third subset that includes the results from blood tests and 2 variables that indicate if the patient was hospitalized and if the patient died. Regarding the subset of health preconditions and treatments, the variables are Asthma [15] (a precondition that affects the breathing capabilities), Diabetes Mellitus [51] (abbreviated as Diabetes, a precondition that is often associated with higher risk patients), Solid Organ Transplant [32] (abbreviated as Transplant, a situation that often means immunosuppression for their sufferers), Chronic Obstructive Pulmonary Disease [17] (abbreviated as COPD, that causes breathing problems), Lymphoma, Neoplasm and Leukemia [29] (associated with immunosuppression), Arterial Hypertension [23] (abbreviated as AHT, a precondition that increases the risk of any pathological condition), Corticosteroids [54] (abbreviated as CCS and associated with immunosuppression), Human Immunodeficiency Virus [53] (abbreviated as HIV and associated with immunosuppression as well), Liver Disease [47] (abbreviated as LD and can affect several mechanisms that help fight against the COVID-19), Chemotherapy within the last 3 months [22] (abbreviated as Chemotherapy, another situation of immunosuppression) and Biological Treatment within the last 3 months [35] (abbreviated as Biological, that has the same impact as the previous variable). In the case of the blood test results, the following variables are included: Creatinine [9] (potential indicator of kidney disorders), Glomerular Filtration Rate [8] (abbreviated as GFR, and potential indicator of inflammation in case it presents abnormalities), D-Dimer Test [64] (related with blood coagulation), Ferritin [24] (a variable related with blood iron), Lactate Dehydrogenase [33] (abbreviated as LDH, and is related with potential evidences of tissue damage), absolute count of lymphocytes (abbreviated as LYMP that, in the case of a low count, could indicate illness) and percentage of lymphocytes [21] (abbreviated as LYMP (pct.)), IL-6 protein test [65] (abbreviated as IL-6, that reports activity of immune response) and C-Reactive Protein [2] (abbreviated as CRP that, in a similar way as with GFR, can indicate inflammation in case it presents abnormalities).

Apart from the clinical features, the used dataset also provides one chest X-ray capture for each patient. The captures present a great variability in terms of resolution, ranging from 1013×1523 to 2098×1523 pixels. These images were obtained as an anterior-posterior projection using 2 different capture devices: the Optima Rx200 and the Agfa dr100E GE. It is important to note that all the chest X-ray images included in this dataset were captured from patients confirmed as COVID-19 positives. Moreover, it is necessary to state that this retrospective study complies with all the necessary regulations. Firstly, it was approved by the local ethics committee of the “Sistema Público de Saúde de Galicia” with the approval number 2020-007. Secondly, informed consent was obtained from all the participants, including the clinical data and the imaging studies. Apart from that, all the data were conveniently anonymized before being released from the CHUAC radiology service, preserving the identity of the patients.

### 3.2 Software and hardware resources

In this section of the manuscript, we present the hardware and software resources necessary to appropriately replicate the methodology. Firstly, the implementation of the methodology was performed using mainly several libraries of machine learning, computer vision and other functionalities, that are detailed in Table 1, using Python 3.8.10 as the programming language. Overall, the xgboost library was used to create, train and evaluate the performance of the machine learning models and torch to extract the deep features from the images. The hardware used is detailed in Table 2. The implementation was executed in the 11^th^ Generation Intel Core i7-11700K CPU, of 3.60 GHz. Moreover, the used operating system was the Ubuntu 20.04.3 LTS (Focal Fossa) with kernel Linux 5.13.0-41-generic. It is important to add that the deep feature extraction process was accelerated using an NVIDIA GeForce RTX 3070 with 8 GB of VRAM. The code used to implement the proposed methodology can be found in the following GitHub repository: https://github.com/Dani-97/multimodal_fusion_covid_19_code. Similarly, the data generated from the experimentation can be found in the repository https://github.com/Dani-97/multimodal_fusion_covid_19_data.

**Table 1:** List of the software requirements needed to implement the methodology of this work.

Name	Version	Description
imblearn	0.0	This library provides tools to deal with imbalanced classification problems.
matplotlib	3.6.0	Matplotlib is a library to visualize the data graphically.
numpy	1.24.3	Numpy provides a set of tools to work with arrays in Python.
pandas	2.0.2	The library pandas allows to perform data analysis.
pillow	9.5.0	Pillow is a library to work with images in Python.
scikit-learn	1.2.2	Library to work with machine learning models in Python.
torch	2.0.1	The torch library enables the use of deep learning models.
torchvision	0.15.2	The library torchvision includes additional functionalities to torch.
xgboost	1.7.5	This library implements the XGBoost classifier.

**Table 2:** List of the hardware specifications used for the experimentation.

Name	Specifications
Motherboard	Gigabyte Z590 AORUS ELITE
RAM	2 x 32GiB DIMM DDR4 Synchronous 3200 MT/s CRUCIAL
	BL32G32C16U4BL.M16FB
Architecture	x86-64
HDD	Seagate IronWolf ST4000VN008-2DR16 (4 TB)
SSD	Western Digital WDS100T1X0E-00AFY (1 TB)

## 4 Methodology

**Figure 1:**
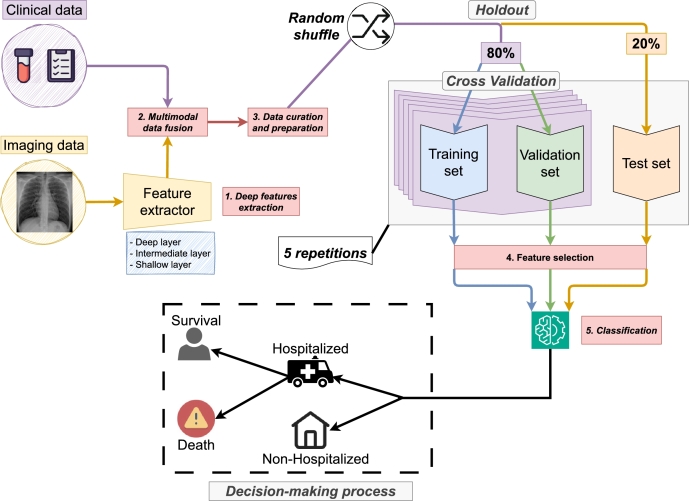
Overview of the methodology proposed in this work that shows the 5 followed sequential steps: deep features extraction, fusion of the features, data curation and preparation, feature selection and classification.

The overview of the efficient fully-automatic methodology proposed in this work can be seen in Fig. 1. This methodology is composed of 5 sequential steps, that are deeply described below. The pipeline starts with a deep feature extraction process, followed by the multimodal data fusion of the clinical and imaging features if corresponds. Once these features are obtained, the data is curated and prepared appropriately for its processing. After that, the score of each feature is calculated, ranking them from the most to the least important, with the aim to perform a process of feature selection. To ensure the robustness of this feature selection process, the data are separated following a cross-validation scheme. Finally, these data are fed to the classifier, to complete the pipeline of the methodology.

**Figure 2:**
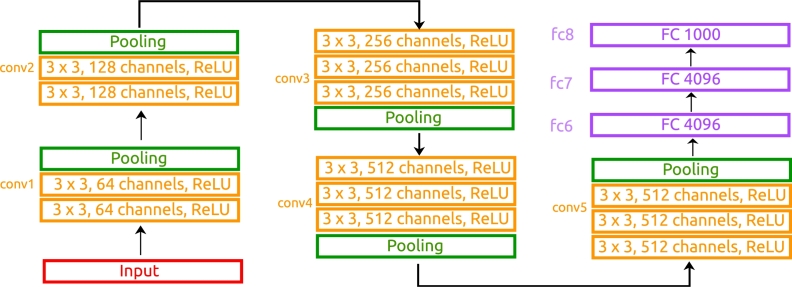
Graphical description of the VGG-16 architecture used to extract the deep features from the images. In particular, fc6, fc7 and fc8 are the layers used to obtain the deep features.

**1**^**st**^**step: Deep features extraction.** The process of deep feature extraction aims to automatically obtain a set of relevant features from the images that can be concatenated with other data sources in a straightforward manner. The use of deep features can be motivated with several advantages. Firstly, it can help to reduce the problem dimensionality, so the same data can be converted to a compressed representation. This dimensionality reduction facilitates the training process and also lightens the computational resource requirements. To this end, we have used a version of the VGG architecture, in particular, the VGG-16 (that is composed of 16 weight layers) [50] whose structure can be seen in Fig. 2. Globally, this architecture is divided in 5 convolutional blocks and 3 fully-connected layers. The first 2 convolutional blocks (denoted as conv1 and conv2) are composed of a sequential scheme of 2 convolutional layers with kernel 3 × 3, each one ending in a ReLU, followed by a pooling layer. The number of channels for each convolutional layer is 64 in conv1 and 128 in conv2. The structure is very similar for the remaining convolutional blocks, but with 3 convolutional layers on each case. Moreover, the number of channels for all the convolutional layers is 256 for conv3, 512 channels for conv4 and 512 channels for conv5. This set of convolutional blocks ends in 3 fully-connected layers that are denoted as fc6, fc7 and fc8, respectively. In particular, fc6 and fc7 have 4096 outputs each, while fc8 has 1000 outputs. In general terms, we can define fc6 as the shallowest layer, fc8 as the deeper layer and fc7 as the intermediate layer. As it happens with all the convolutional neural network architectures, the shallowest layers contain more local features, with a finer grain, while the deeper layers contain more global features. This range of different layers allows the understanding of which is the most appropriate feature level to better characterize the problem. In addition to the original VGG-16 architecture, we concatenate a ReLU layer after each fc6, fc7 and fc8 with the aim to avoid negative activations and threshold them to 0. Therefore, some imaging features will provide no information to the system for any of the samples. In those cases, given that the variance will be 0, the affected features will be removed from the dataset. After this filtering process, a total of 3645 features will be obtained from the fc6 layer, 3243 from the fc7 layer and 925 from the fc8 layer.

**2**^**nd**^**step: Multimodal data fusion.** Each sample of the dataset is expressed as a feature vector, with one vector for the clinical features and another one for the deep features extracted from the images. In particular, we consider 3 different approaches, with 2 baselines (*i.e.*, with a single data source) and another approach that fuses both data sources. These approaches are described as can be seen below:

• **Approach I (only clinical data):** in this case, only the vector with the clinical features is considered. The aim of this approach is to provide a baseline with the same specifications that were defined in [36]. This means that no additional data sources will be added (only the same 28 variables that were used in the previous work will be considered).
• **Approach II (only imaging data):** for the second approach, only the vector of the imaging features is considered. Training with only imaging data aims to provide a second baseline, with the new data source that is contemplated in this work.
• **Approach III (clinical data + imaging data):** in this last case, the vector of clinical features is fused with the vector of deep features. The objective of the approach is to compare the performance of the multimodal data fusion with the 2 individual baselines previously discussed.

The aim of proposing 3 different approaches is to compare the contexts where a single data source is considered (only clinical data or only imaging data) to see the individual contribution of each data type and the situation where both data sources are used together, to evaluate the performance improvement that this implies for the classification model. It is important to note that, for this methodology, the multimodal data fusion stage is conducted by the means of a concatenation between both feature vectors.

**3**^**rd**^**step: Data curation and preparation.** Regarding this step of the methodology, we have followed the same criteria as defined in [36]. Initially, it is necessary to perform some checks and processing to ensure that the data have the appropriate quality. Firstly, to deal with the situations of missing values, the discrete variables (*i.e.*, those that refer to preconditions that the patients have or treatments that they may be taking) are 0-padded. In the case of the numerical variables, the missing values are padded with -1. Other issue related with data is the fact that the distribution of variables may be considerably different, an aspect that could negatively impact the performance of the classification models. For that reason, we have applied a standardization to the features, to ensure that all the variables have a mean of *μ*= 0 and a standard deviation of *σ*= 1 and that they have similar ranges. In this way, we avoid the situation where some features are given more weight than others in an undesired manner. This is specially important given that some features are categorical, others are numerical and that the ranges are considerably different.

**4**^**th**^**step: Feature selection.** When working with the provided dataset, several issues can appear. Firstly, some features could be redundant or even useless, an aspect that makes the classification model performance drop, given that it would find no correlation between those features and the desired output. Moreover, the clinical environments usually lack of high performance computing architectures, necessary to execute the deep learning algorithms, that require special hardware and have expensive memory requirements. To avoid such situations, the feature selection algorithms aim to find a subset of features that characterizes the problem without losing relevant information but in a compressed representation. This can significantly reduce the previously-mentioned memory requirements, making it possible to execute the algorithms in more traditional hardware. Ultimately, this allows implementing an efficient method that can fit the clinical environments. The process starts giving a score to each feature, according to the criteria of a specific algorithm. This algorithm will give a high value when it exists a high correlation between a feature and the target variable and a low value when the correlation is weak. Consequently, that value will be used as the score of the studied feature. For these purposes, the selected algorithm was the Mutual Information method [56]. Given two random variables, the Mutual Information algorithm gives a high value when there is a strong correlation between them and a low value when there is a weak correlation. For the context of this work, this algorithm will measure how much information a variable *X* (that denotes an individual feature) provides about another variable *Y* (that denotes the target for the model) or, based in the idea of entropy, the higher the value, the higher will be the reduction of uncertainty when using the variable *X* to determine the value of *Y*. The final objective of using Mutual Information is to calculate the previously-mentioned score for each individual feature that will be then used to build the ranking. To express the equation of Mutual Information, it is necessary to first define the entropy of a variable *X*, denoted as H(X). This expression can be seen in Equation (1):

(1) $$H(X)=-\sum_{i=1}^{n}P(x_{i})\log P(x_{i})$$

Nevertheless, this expression only considers the entropy related to one variable, and that is why it is necessary to define a second expression, the conditional entropy, denoted as H(X|Y). This allows correlating the 2 variables *X* and *Y*. Then, this statistical formula can be expressed as in Equation (2).

(2) $$H(X|Y)=-\sum_{i,j}P(x_{i},y_{j})\log\frac{P(x_{i},y_{j})}{P(y_{j})}$$

Finally, these 2 expressions can be merged together to compute the value of mutual information *I* as is shown in Equation (3):

(3) I(X,Y)=H(X)−H(X|Y)

Once the feature ranking is obtained, we select the top *N* features (*i.e.*, the *N* features with the highest score). The optimal value of *N* is unknown and different for each context, as it depends on aspects like the kind of features that are being considered (clinical data, imaging data or both) or the layer of the VGG-16 architecture used to obtain the deep features, among others. Therefore, the selection of the optimal value of *N* will be performed as a part of the experimental process.

**5**^**th**^**step: Classification.** In this step of the methodology, we train the XGBoost model to estimate the risk of the COVID-19 patients in the 2 studied scenarios [10]. Regarding the training parameters of this model, we limited the maximum depth of the trees to 1 and we have chosen to conduct a random subsampling of 0.5 of the training subset before growing the trees. The selection of these parameters aims to reduce the complexity of the model and increase the randomness within the training process to reduce the risk of overfitting. The input data is randomly split in a holdout fashion, giving an 80% of the samples for training and the remaining 20% for test. Apart from that, the 80% of samples used for training are split following a 5-fold cross-validation scheme, that obtains 5 pairs of training and validation sets.

**Figure 3:**
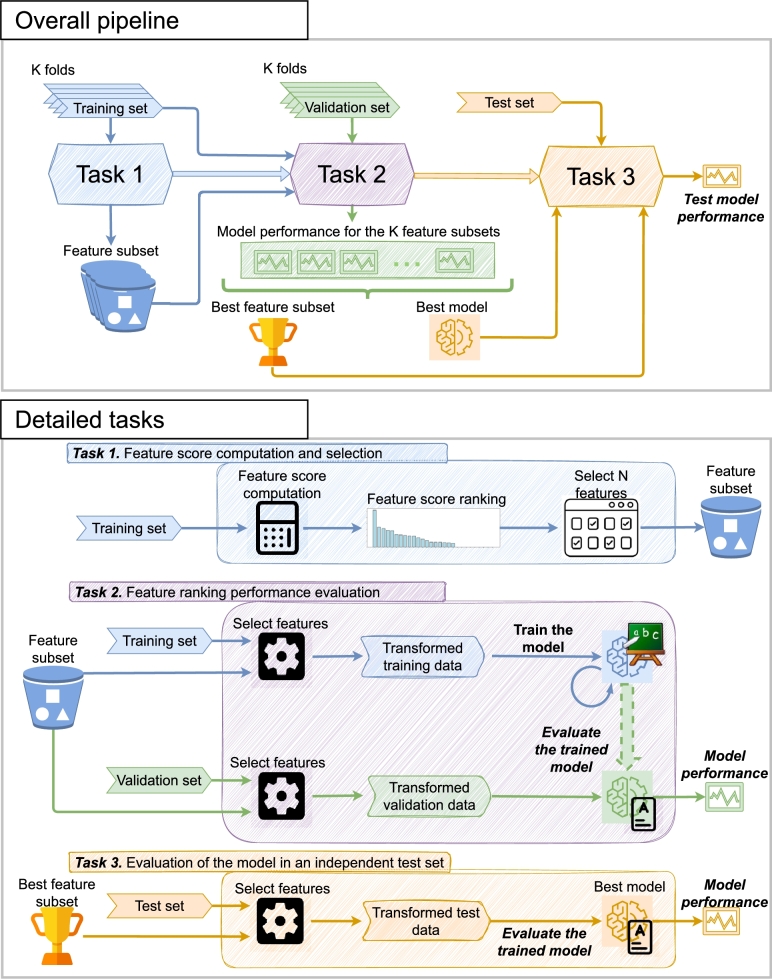
Description of how the feature selection process is integrated with classification, that includes an overall depiction of the pipeline and a detailed definition of each step involved.

At this point, it is important to detail how the feature selection process is integrated with the classification, an aspect that is depicted in Fig. 3. This integration is performed by the means of three tasks. The first task (feature score computation and selection) computes the score of each feature to then build the feature ranking. From this feature ranking, *N* features are selected. Having this feature subset, in the second task (feature ranking performance evaluation) the classification model is trained with a transformed version of the training set, that will only include the selected features. Then, during inference time, the trained model is evaluated with the validation set. The described process is performed 5 times, one for each fold of the cross-validation split. After that, the performance metrics will be available for the 5 feature subsets, from which the best subset and the best model are selected (*i.e.*, those from the scenario that obtained the highest metrics). In the task 3, with the remaining 20% of samples for the test set, an independent evaluation of the best trained model with the best feature subset is performed. Finally, it is necessary to remark that the initial holdout step is repeated 5 times, with its corresponding cross validation process and the test stage, to provide a better insight of the actual performance. This allows to report the mean and the standard deviation of all the validation metrics.

Apart from the data splitting, it is relevant to mitigate the problem of imbalance. To that end, in this methodology, we have considered a random oversampling. Given a majority class of *N* samples and a minority class of *M* samples, this oversampling consists in a random selection of N−M samples from the minority class that are then reincorporated to that class itself. In this way, we ensure that both classes have the same cardinality while only using the data that is already available.

### 4.1 Description of the scenarios

This study is centered around two critical scenarios, each one utilizing a specific version of the original CHUAC dataset, a resource meticulously designed specifically for this study. These scenarios are carefully selected to reflect the pressing challenges faced by healthcare professionals in managing COVID-19. The first scenario examines the estimation of hospitalization risk, a crucial factor in healthcare resource allocation and patient management. The second scenario tackles the estimation of mortality risk, a stark reality in the fight against the pandemic. In these distinct yet intertwined scenarios, we evaluate the model's performance, offering insights that could potentially improve patient care and clinical decision-making processes.

**Scenario I. Risk of hospitalization (Non-Hospitalized/Hospitalized).** The first scenario contemplates the situation where a COVID-19 patient comes to an emergency room. When this occurs, the individual may be attended on site or hospitalized, depending on the severity and risk. Therefore, in this case, we evaluate whether a patient requires hospitalization (in which case it will be defined as Hospitalized) or can be released after coming to an emergency room (defined as Non-Hospitalized).

**Scenario II. Risk of death (Survival/Death).** In case that a patient is hospitalized, we contemplate 2 possible outcomes. If the patient overcomes the disease, then it will be considered as a Survival. Otherwise, if the patient fails to overcome the disease, it will be considered as a Death.

### 4.2 Evaluation metrics

To evaluate the performance of the classification models, we provide a report with some of the most relevant metrics used in the state-of-the-art for classification problems. Denoting TP as True Positives, TN as True Negatives, FP as False Positives and FN as False Negatives, we compute the following metrics as stated in their corresponding equations: accuracy (Equation (4)), recall (Equation (5)), precision (Equation (6)), specificity (Equation (7)), F1-Score (Equation (8)) and Matthews Correlation Coefficient (Equation (9), abbreviated as MCC).

(4) $$Accuracy=\frac{TP+TN}{TP+TN+FP+FN}$$

(5) $$Recall=\frac{TP}{TP+FN}$$

(6) $$Precision=\frac{TP}{TP+FP}$$

(7) $$Specificity=\frac{TN}{TN+FP}$$

(8) $$F1-Score=\frac{2⁎Recall⁎Precision}{Recall+Precision}$$

(9) $$MCC=\frac{TN\times TP-FN\times FP}{\sqrt{\left(TP+FP)(TP+FN)(TN+FP)(TN+FN\right)}}$$

Moreover, we also provide a global evaluation of the performance computing the area under the ROC (Receiver Operating Characteristic) curve, denoted as AUC-ROC. This evaluation metric calculates the True Positive Rate (equivalent to recall in this work), denoted as TPR, and the False Positive Rate (that is equivalent to 1-specificity in this work), denoted as FPR, with different operation points. This metric is obtained as expressed in Equation (10).

(10) $$AUC-ROC=\int_{0}^{1}TPR\cdot d(FPR)$$

## 5 Results and discussion

In this section, we delve into the outcomes and discussion surrounding our exhaustive experimental process. We carried out a comprehensive assessment under two relevant scenarios: evaluating the risk of hospitalization and the risk of mortality. Our approach was threefold - we firstly assessed using only clinical data, secondly, we examined utilizing imaging data, and lastly, we combined both data types to explore the data fusion approach.

Furthermore, to understand the complexity of the data, we scrutinized three specific layers of the VGG-16 architecture. This inspection aimed at capturing both basic and complex features, from elementary visual aspects like edges and colors to more intricate patterns and structures. The thorough analysis allows us to select the most representative features pertinent to the problem at hand, subsequently improving the optimality and efficiency of the solution.

Our study commenced with the examination of the distribution of the clinical variables, distinguishing between the discrete and numerical variables. We then traced the evolution of the F1-Score with respect to the number of selected features, and drew a comparative analysis between the performances of each approach and the different layers of VGG-16. From that evolution, the highest-performing results of each approach are obtained and compared with several classification metrics, also reporting the number of features of each type that were used. Considering that the combination of a cross-validation and a holdout scheme will provide several highest-performing feature subsets, the number of clinical and imaging variables will be the average among repetitions.

In the concluding part of our analysis, we created a ranking system to determine the clinical variables' significance, which further assisted in identifying the optimal resource in multimodal scenarios. Given that each experiment is repeated 5 times, this will provide 5 different highest-performing feature subsets. Consequently, the reported rankings will be based on the mean scores given for each feature among the 5 repetitions.

The results for the first scenario, i.e., estimation of the hospitalization risk, are elaborated in Subsection 5.1. The findings for the second scenario, i.e., the estimation of the mortality risk, are explicated in Subsection 5.2. In the final subsection, 5.3, we juxtapose the performances obtained in this research with those of other state-of-the-art works with similar objectives.

### 5.1 Scenario I. Estimation of non-hospitalized/hospitalized

**Figure 4:**
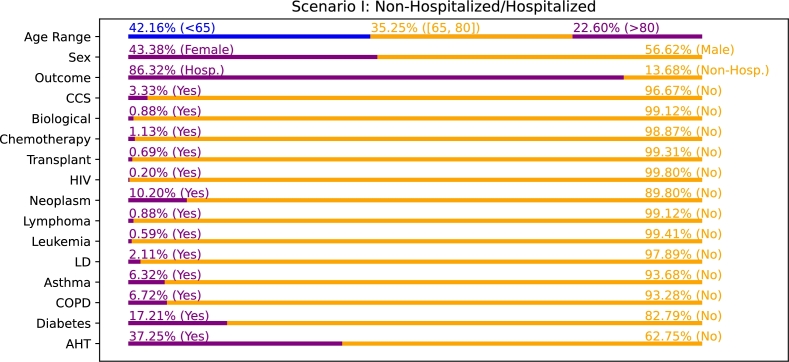
Distribution of values for the discrete variables in scenario I (Non-Hospitalized/Hospitalized).

For the version of the dataset in this first scenario, the distribution of the discrete variables can be seen in Fig. 4. In particular, the Age Range shows a great amount of patients within the range of [65, 80] (in particular, 35.25%), as a significant portion of the COVID-19 patients who require medical attention are often of an advanced age (patients that are considered of a higher risk). Regarding the variable Sex, it is interesting to remark that the dataset is quite balanced between Males and Females, with a 56.62% and a 43.38%, respectively. Finally, regarding the variable Outcome, an 86.32% of the patients were hospitalized and the 13.68% of patients were released before requiring hospitalization. This important imbalance is caused by the fact that most of the COVID-19 patients who require medical attention can recover at home, without any further testing. Only those patients that present a more severe form of the disease are asked to perform blood tests and/or chest X-ray studies.

Regarding the rest of the discrete variables, the vast majority present an important imbalance. In particular, there are less than 4% of patients that present the preconditions HIV, Lymphoma, Leukemia and LD, that require Chemotherapy, CCS, Biological or that have a Transplant. The variables with the biggest incidence are Asthma, COPD, Neoplasm, Diabetes and AHT. It is interesting to note that the percentage of patients that present Diabetes is 17.21% and the percentage for AHT is 37.25%. This is remarkable of the great incidence that these preconditions have in elderly patients. Regarding the numerical variables, their distributions are shown in Table 3 in terms of the first quartile, median and third quartile. These distributions' ranges demonstrate to be notably different, a heterogeneity that increases when including the imaging features. The described heterogeneity makes more necessary to apply normalization on data, and make these data work in the same ranges.

**Table 3:** Distribution of the numerical clinical variables in terms of median, *Q*_1_ and *Q*_3_.

Feature	Unit	Median (*Q*_1_ - *Q*_3_)
BMI	*Kg*/*m*^2^	29.76 (26.71 - 32.93)
Creatinine	*mg*/*dL*	0.94 (0.76 - 1.19)
CRP	*mg*/*L*	5.47 (1.69 - 11.73)
D-Dimer	*ng*/*mL*	748.00 (445.00 - 1286.00)
Ferritin	*ng*/*mL*	415.50 (152.00 - 820.75)
GFR	*mL*/*min*	78.72 (52.46 - 111.07)
Height	*cm*	163.00 (156.00 - 170.00)
IL-6	*ng*/*L*	18.70 (7.60 - 46.90)
LDH	*U*/*L*	264.00 (202.00 - 362.00)
LYMP	10^9^/*L*	1.00 (0.70 - 1.45)
LYMP (pct.)	%	17.30 (11.30 - 25.60)
Weight	*Kg*	80 (68.95 - 90.00)

**Figure 5:**
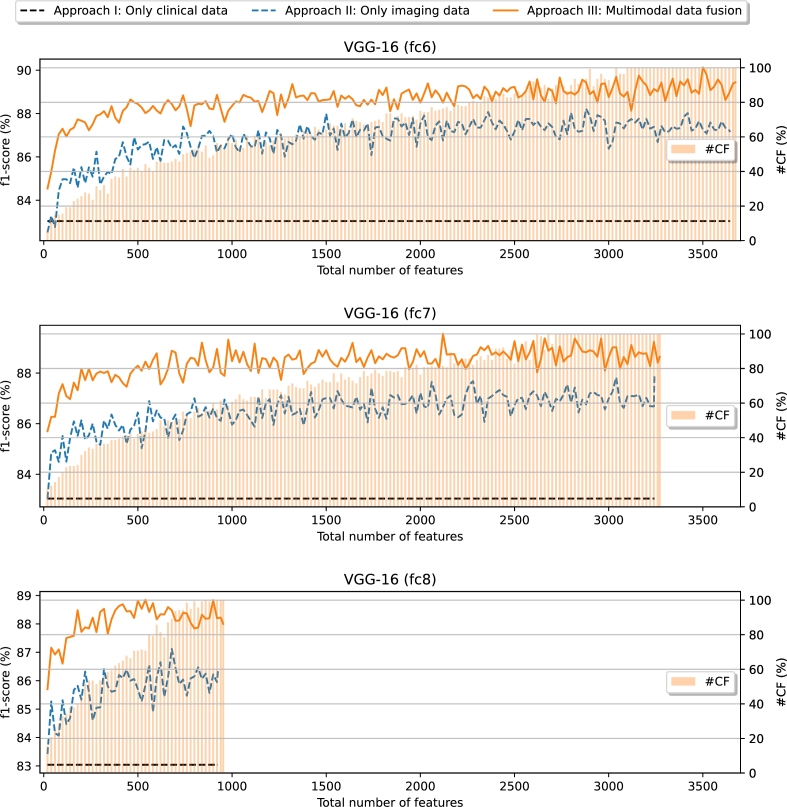
Performance evolution for the scenario I (Non-Hospitalized/Hospitalized) using the imaging features obtained from the 3 considered VGG-16 layers (fc6, fc7 and fc8). #CF: average number of clinical features (only applies for approach III).

The performance evolution in terms of the F1-Score can be seen in Fig. 5, regarding the 3 approaches and the 3 used VGG-16 layers. In particular, we have studied the performance with a great amount of different numbers of used features, starting from 20 and increasing by 20 on each step until reaching the total amount of available features. This evolution depicts a notable improvement of the performance with approach II and approach III over approach I. Moreover, there is always an important gap between approach III and approach II, being the first one at the top. Overall, considering the 3 VGG-16 layers, an important improvement is even noticeable for the considered minimum amount of features, 20. With the VGG-16 fc6 layer, the F1-Score starts from a very competitive performance (above 84%) and obtains the highest performance when getting close to 3500 features in the case of the approach III. On the other hand, for approach II, the performance seems to stabilize around 2000 features onward. It is interesting to remark that a competitive performance (higher than 88% for approach III and 86% for approach II) can be achieved with less than 60% of the 28 clinical features in average. Regarding the VGG-16 fc7, a competitive performance is achieved earlier in comparison with VGG-16 fc6, going past 88% for approach III and 86% for approach II, with less than 40% of the total amount of clinical features in average. Moreover, it is interesting to see that the performance shows a trend of stabilization between 2000 and 2500 features in both approaches II and III. The trend of stabilization can be seen earlier in the case of VGG-16 fc8, achieved around 500 features in both approaches with imaging features (II and III). Once again, similarly as with the previous VGG-16 layers, the performance goes past 88% of F1-Score when using an average of less than 60% of the clinical features for approach III and past 86% for approach II. From these results, some conclusions can be extracted. Firstly, there are evidences of the great contribution that the imaging features bring to the model performance. This is explained by the competitive performance achieved by the approach II, where only imaging features are considered, outperforming the approach I with a notable margin.

In addition, the highest-performing results obtained in this scenario for each approach and VGG-16 layer (*i.e.*, those with the highest AUC-ROC) can be seen in Table 4, comparing approaches II and III with approach I. From those results, some interesting discussions can be extracted. Firstly, the approach II shows an important improvement over approach I in terms of accuracy, F1-Score and recall. On the other side, there are some performance drops in terms of MCC, specificity (that is compromised by the model to improve the recall) and AUC-ROC. Interestingly, the global performance is competitive in comparison with approach I, showing that imaging features are notably relevant to determine the risk of a patient to be hospitalized. The second discussion that can be extracted is that, when the model is solely trained with image data, a great amount of imaging features is needed to present a high performance, regardless of the layer from where the deep features were extracted.

**Table 4:** Comparison of the results among approaches for the scenario Non-Hospitalized/Hospitalized (#CF: average number of clinical features; #IF: average number of imaging features). Approach I: only clinical data. Approach II: only imaging data. Approach III: multimodal data fusion.

Approach	**Table:** VGG-16 layer	#CF	#IF		Accuracy	MCC	F1-Score	Precision	Recall	Specificity	AUC-ROC
I	N/A	28.0	0.0	*μ*	74.22%	0.3859	83.04%	95.75%	73.38%	79.59%	0.8398
				*σ*	1.39%	0.0326	1.20%	1.45%	2.35%	7.22%	0.0240

II	fc6	0.0	2900.0	*μ*	80.00%	0.3220	87.89%	91.88%	84.25%	53.70%	0.7621
				*σ*	0.88%	0.0313	0.55%	0.78%	1.38%	4.03%	0.0220
	fc7	0.0	2460.0	*μ*	78.74%	0.2962	87.06%	91.56%	83.02%	52.57%	0.7586
				*σ*	0.34%	0.0241	0.28%	1.32%	1.41%	5.93%	0.0206
	fc8	0.0	820.0	*μ*	78.02%	0.3053	86.47%	91.99%	81.61%	55.90%	0.7508
				*σ*	1.27%	0.0153	0.94%	0.99%	2.08%	4.51%	0.0231

III	fc6	28.0	3472.0	*μ*	83.57%	0.4260	90.13%	93.39%	87.11%	61.74%	0.8297
				*σ*	1.42%	0.0277	0.92%	0.56%	1.95%	1.73%	0.0213
	fc7	16.4	963.6	*μ*	82.37%	0.4065	89.32%	93.36%	85.66%	62.20%	0.8376
				*σ*	1.24%	0.0307	0.82%	1.09%	2.10%	5.56%	0.0114
	fc8	19.8	460.2	*μ*	80.82%	0.3972	88.21%	93.71%	83.36%	65.13%	0.8452
				*σ*	1.86%	0.0508	1.18%	0.84%	2.24%	4.86%	0.0133

When visualizing the results for the approach III, several performance improvements can be seen in comparison with both approach I and II. Particularly, all the accuracy values for the 3 VGG-16 layers improve both previous approaches. The same conclusion can be obtained for F1-Score. In the case of precision, the greatest performance is achieved by approach I, but approach III improves the approach II in all the cases. Regarding the recall, both approaches II and III outperform the approach I. Moreover, when comparing approach II with approach III considering the same VGG-16 layer (*i.e.*, comparing fc6 in approach II with fc6 in approach III and so on), the approach III is always the one with the highest recall. In the case of specificity, the highest value is achieved by approach I but, in a similar line as with precision, the approach III outperforms approach II in this metric. Finally, when analyzing the performance in terms of AUC-ROC, it can be seen that the approach I obtains a greater performance than approach II. However, the approach III shows very similar metrics (being the lowest AUC-ROC 0.8297 for the VGG-16 fc6 layer against 0.8398 for approach I) while, when using the VGG-16 fc8 layer, the performance goes up to 0.8452. One of the most important aspects that must be globally highlighted about the obtained results is that approaches II and III show a great improvement of the recall and F1-Score while compromising the specificity and the precision. This imbalance of the metrics showcases that different types of features have a different impact on performance. Particularly, the clinical variables seem to provide a more balanced way to model the problem (thus having a lower recall but smaller trade-off between recall and specificity), while the imaging features seem to improve the capabilities of the positive class prediction while having a negative impact on specificity. Despite that, the MCC is higher in the case of the approach III, while the precision is very similar and the F1-Score is higher.

Regarding the number of features of each type that are needed, several interesting discussions can be obtained. Firstly, the highest AUC-ROC is achieved in approach I when using the whole amount of 28 clinical features. Secondly, talking about approach II, for each individual case, the average amount of needed imaging features is always over 75% of the total number (2900.0 of 3645 in the case of VGG-16 fc6, 2460.0 of 3243 for VGG-16 fc7 and 820.0 of 925 for VGG-16 fc8). In the case of the approach III, it is shown that fusing both data sources can help to reduce the needed amount of each type of features to obtain a high performance in some cases. Interestingly, when using the VGG-16 fc6 layer, the highest AUC-ROC is achieved when using the whole amount of clinical features (an average of 28.0 features) and an even greater percentage of imaging features (an average of around 95%, 3472.0 of 3645) in comparison with the same circumstances of approach II. Nevertheless, the reduction in number for both data sources with VGG-16 fc7 and fc8 is notable, needing only an average of 16.4 clinical features and 963.6 imaging features as well as 19.8 clinical features and 460.2 imaging features, respectively. Consequently, from the approach III it can be obtained that the imaging features provide a great amount of information to estimate the risk of hospitalization in COVID-19 patients, making the clinical variables to lose relevance, as some of them can be discarded without compromising the performance. When discussing the impact of using shallower or deeper VGG-16 layers regarding the global performance (taking the AUC-ROC metric as reference), it is concluded that the approach II is more benefited from using the shallower layer (VGG-16 fc6, obtaining the highest AUC-ROC of 0.7621) while the approach III obtains the highest performance with the deeper layer (VGG-16 fc8, obtaining the highest AUC-ROC of 0.8452). From this, it can be extracted that, when using only imaging to estimate the risk of hospitalization, the local features give more information than global features. Nevertheless, the opposite happens when combining the information with clinical data, as global features gain importance over local features.

**Figure 6:**
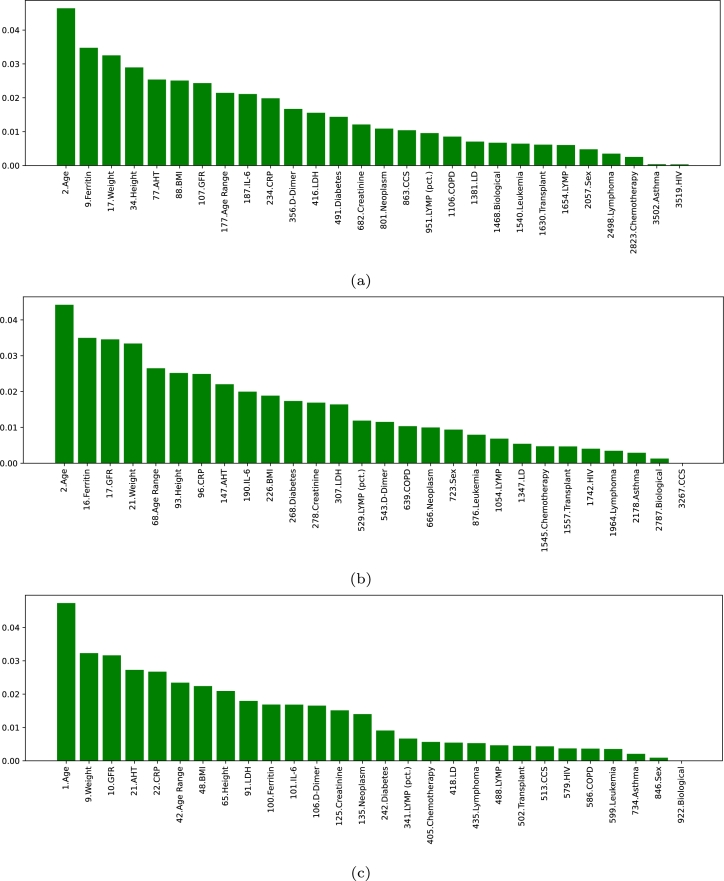
Ranking of the clinical features in the first scenario (Non-Hospitalized/Hospitalized), indicating their global position (*i.e.*, considering both the clinical data and the imaging data) regarding the 3 different created datasets (a) Clinical data + Imaging data (VGG-16 fc6). (b) Clinical data + Imaging data (VGG-16 fc7). (c) Clinical data + Imaging data (VGG-16 fc8).

Furthermore, the ranking of the features in scenario I is shown in Fig. 6. As it can be seen, regardless of the VGG-16 layer from where the deep features are extracted, the variable Age is ranked among the most important features in all cases. In the particular case of the VGG-16 fc6 layer, the Age is placed in second position, while other clinical variables are also ranked within the top 100. To be precise, this is the case of Ferritin (position 9), Weight (position 17), Height (position 34), AHT (position 77) and BMI (position 88). For VGG-16 fc7, the position of Age, Ferritin and Weight follows a similar trend as in the previous case (with the positions 2, 16 and 21, respectively). Moreover, GFR is also given a great importance in this case (position 17), while the other clinical variables within the top 100 are Age Range (position 68), Height (position 93) and CRP (position 96). In the case of VGG-16 fc8, the trend of the clinical variables is to be ranked higher than in the previous cases. In particular, several features are ranked within the top 25, with Age placed as the most important feature overall, Weight placed in position 9, GFR in position 10, AHT in position 21 and CRP in position 22. From then onward, there are some more variables within the top 100: Age Range (position 42), BMI (position 48), Height (position 65), LDH (position 91) and Ferritin (position 100), being IL-6 placed very close (in position 101). The higher ranking of the clinical variables is mainly motivated by two factors. Firstly, the number of deep features that can be extracted from the layers is progressively smaller, making it easier for clinical features to rank higher. The other aspect is that the fc6 layer presents the lowest-level features and the fc8 the highest-level features, while fc7 is in the middle of both. Therefore, it is plausible that, when using the fc6 layer, the model needs more low-level features, while in the case of fc8, the number of imaging features is smaller, because they contain more high-level information.

### 5.2 Scenario II. Estimation of survival/death

**Figure 7:**
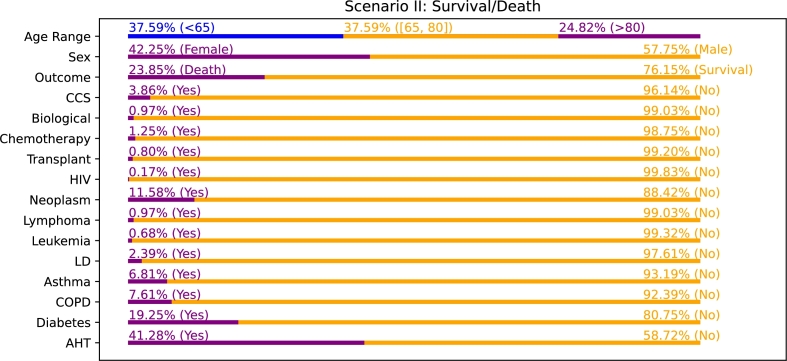
Distribution of values for the discrete variables in scenario II (Survival/Death).

The Fig. 7 shows the distribution of the discrete variables in this scenario II. There, regarding the variable of Age Range, it can be seen the great amount of patients in the range [65, 80] (in particular, 37.59% of the patients), that in this case is very close to the percentage within the range <65 (with a percentage of 37.59%). This is significant of the important number of hospitalized patients that belong to the elderly cohort. Regarding the variable Sex, the balance is very similar as in scenario I. Finally, the outcome is, in the same way as in scenario I, considerably imbalanced, as the 76.15% of the hospitalized patients survived and the 23.85% died. The distribution of the remaining discrete variables is very similar to that reported in scenario I. Once again, the variables CCS, Biological, Chemotherapy, Transplant, HIV, Lymphoma, Leukemia and LD have less than 4% of positive samples each, while Asthma, COPD, Neoplasm, Diabetes and AHT have an incidence greater or equal than 6.81%. In this case, Diabetes and AHT have a slightly higher incidence, with a 19.25% and a 41.28%, respectively.

Finally, the distribution of values in the case of the numerical variables is shown in Table 5. Closely related with the distribution reported in scenario I, the ranges of each variable are notably different, an aspect that is even more pronounced when adding the imaging features.

**Table 5:** Distribution of values for the numerical variables in terms of *Q*_1_, *Q*_3_ and the Median for the dataset of the scenario II (Survival/Death).

Feature	Unit	Median (*Q*_1_ - *Q*_3_)
BMI	*Kg*/*m*^2^	29.76 (26.67 - 32.87)
Creatinine	*mg*/*dL*	0.94 (0.77 - 1.20)
CRP	*mg*/*L*	5.95 (1.91 - 12.25)
D-Dimer	*ng*/*mL*	755.50 (459.50 - 1311.00)
Ferritin	*ng*/*mL*	424.00 (157.00 - 831.50)
GFR	*mL*/*min*	78.72 (52.35 - 110.56)
Height	*cm*	163.00 (156.00 - 170.00)
IL-6	*ng*/*L*	19.05 (7.60 - 47.42)
LDH	*U*/*L*	272.50 (209.00 - 372.00)
LYMP	10^9^/*L*	1.00 (0.70 - 1.45)
LYMP (pct.)	%	16.90 (10.80 - 25.30)
Weight	*Kg*	80.00 (68.85 - 90.00)

**Figure 8:**
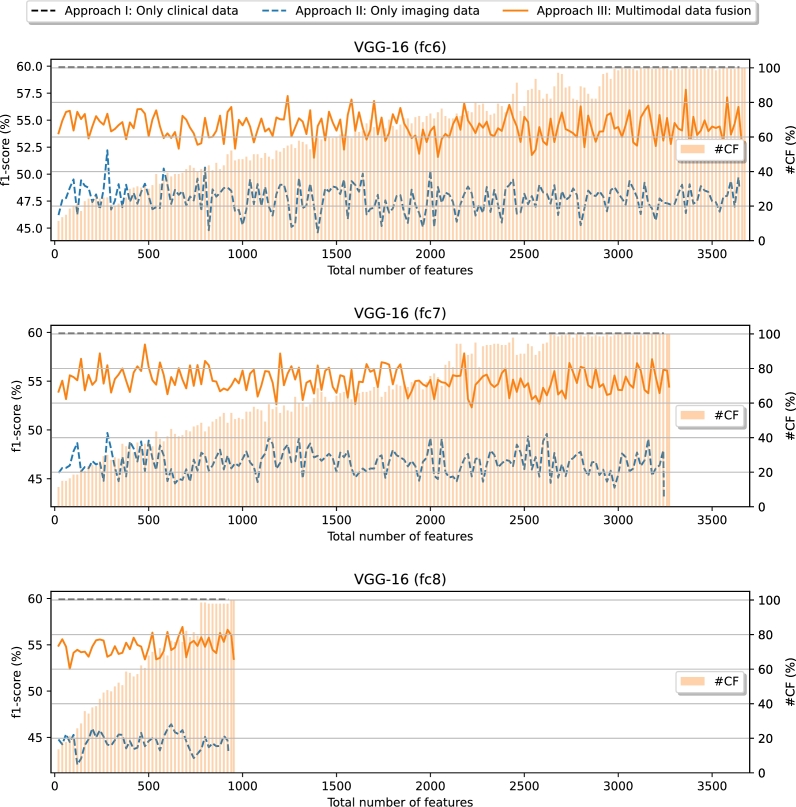
Performance evolution for the scenario II (Survival/Death) using the imaging features obtained from the 3 considered VGG-16 layers (fc6, fc7 and fc8). #CF: average number of clinical features (only applies for approach III).

The Fig. 8 presents the evolution of the performance as the total number of features is increased in terms of F1-Score, starting from 20 features and adding 20 more on each step until reaching the whole amount available. The general conclusion that can be extracted is that the approach II obtains a considerably lower performance in comparison with approach I and approach III. This applies to all the layers of the VGG-16 architecture. In fact, when looking the results obtained with the imaging features extracted from VGG-16 fc6, the highest performance is achieved very early (with less than 500 features) and the trend of F1-Score seems to be going slightly downward from there. In general, for the rest of the cases and the approach III, the trend of the F1-Score seems to be always considerably plain and stable from the start to the finish, with some specific points where the performance reaches peaks. Interestingly, in this scenario, the approaches II and III seem not to bring an improvement over approach I. This is indicative that the imaging features are less powerful than in the previous scenario, showcasing how the same data source can present evidences of certain risks (in the case of scenario I, the capability of estimating the risk of hospitalization with imaging features) while not presenting evidences of other different risks (in the case of scenario II, the reduced capability of estimating the risk of death with imaging features and the greater capability of the clinical features). Consequently, the fusion of both data sources can mean no improvement and even a worsening with respect to approach I (as using imaging features can have a negative impact on the performance despite also using clinical features with a greater outcome estimation ability).

From those F1-Score evolutions, Table 6 extracts the highest-performing results (*i.e.*, those with the greatest AUC-ROC) for each approach and VGG-16 layer in this second scenario. When comparing the approach II with the approach I, we can see a general drop in performance for all the metrics, following the same coherence as when discussing the F1-Score evolution. In the case of the approach III, similar conclusions can be extracted (lower MCC, F1-Score, precision, recall and AUC-ROC in comparison with approach I), although an improvement of accuracy (going from 76.03% ± 1.44% to the highest value of 77.42% ± 2.50%) and specificity (raising from 77.82% ± 1.79% to the highest value of 81.03% ± 2.02%) is appreciated. Furthermore, there is an improvement over approach II, being outperformed for all metrics. Globally, it is interesting to remark that, the trade-off between recall and specificity is lower in the case of the approach I and biased toward one of the classes in approaches II and III, following a similar trend as in scenario I. However, in this scenario I, the negative predictive capabilities of the classifier are improved while compromising the recall, the precision and, consequently, the F1-Score. Therefore, the clinical variables, once again, demonstrate to have a more balanced way to represent the problem while the imaging features have a better understanding of the patterns that define the negative class (making the classifier to perform better in terms of specificity).

**Table 6:** Comparison of the results among approaches for the scenario Survival/Death (#CF: average number of clinical features; #IF: average number of imaging features). Approach I: only clinical data. Approach II: only imaging data. Approach III: multimodal data fusion.

Approach	**Table:** VGG-16 layer	#CF	#IF		Accuracy	MCC	F1-Score	Precision	Recall	Specificity	AUC-ROC
I	N/A	28.0	0.0	*μ*	76.03%	0.4462	59.88%	51.96%	71.12%	77.82%	0.8300
				*σ*	1.44%	0.0287	2.37%	3.85%	3.53%	1.79%	0.0158

II	fc6	0.0	2440.0	*μ*	72.83%	0.3197	49.49%	44.12%	56.84%	77.84%	0.7620
				*σ*	1.03%	0.0224	2.26%	4.00%	2.76%	1.85%	0.0190
	fc7	0.0	920.0	*μ*	71.71%	0.2900	47.38%	42.13%	54.50%	76.91%	0.7471
				*σ*	1.78%	0.0443	4.43%	4.68%	5.85%	3.09%	0.0217
	fc8	0.0	900.0	*μ*	69.36%	0.2537	45.18%	39.12%	53.89%	74.12%	0.7300
				*σ*	1.95%	0.0132	1.17%	2.52%	3.09%	3.10%	0.0230

III	fc6	28.0	3552.0	*μ*	76.92%	0.4249	57.11%	50.76%	65.75%	80.46%	0.8198
				*σ*	2.06%	0.0556	4.20%	4.38%	6.39%	1.77%	0.0308
	fc7	24.4	2155.6	*μ*	77.42%	0.4345	57.85%	51.68%	66.05%	81.03%	0.8285
				*σ*	2.50%	0.0547	3.68%	4.20%	5.14%	2.02%	0.0210
	fc8	27.4	872.6	*μ*	76.25%	0.4130	56.28%	49.76%	65.37%	79.64%	0.8228
				*σ*	1.94%	0.0463	3.61%	4.56%	5.96%	2.68%	0.0285

Regarding the number of features of each class that are needed, the approach I obtains the highest performance with the whole amount of clinical features. In the case of the approach II, a great amount of imaging features is needed from VGG-16 fc6 and fc8 layers (more than half of the available features in each case) while a much smaller relative amount is necessary when considering the VGG-16 fc7 layer (less than 30% of the available features, with 920.0 of 3243). While analyzing the approach III, it is interesting to discuss that a great amount of features of each type is necessary to obtain a higher performance than in approach II. In particular, when using the VGG-16 fc6 layer, the highest value of AUC-ROC is achieved with an average amount of 28.0 clinical features and an average close to the whole amount of imaging features (3552.0 of 3645, bigger than 2440.0 in approach II). For the VGG-16 fc7 layer, the average amount of clinical variables goes down to 24.4 and the average number of imaging features goes down to 2155.6 (much higher than the 920.0 imaging features needed in the same context of approach II). Finally, in the case of the VGG-16 fc8 layer, the average amount of clinical features is 27.4 and the average amount of imaging features is 872.6. Regarding the analysis of the most important VGG-16 layer, it is interesting to remark that the features extracted from the shallower layer (fc6) obtain the highest performance in approach II (0.7620 ± 0.0190 of AUC-ROC) and from the fc7 layer in approach III (0.8285 ± 0.0210 of AUC-ROC). Therefore, the results suggest that, to estimate the risk of death, the shallower layers (those that provide the most local features) are the ones that tend to give more information.

**Figure 9:**
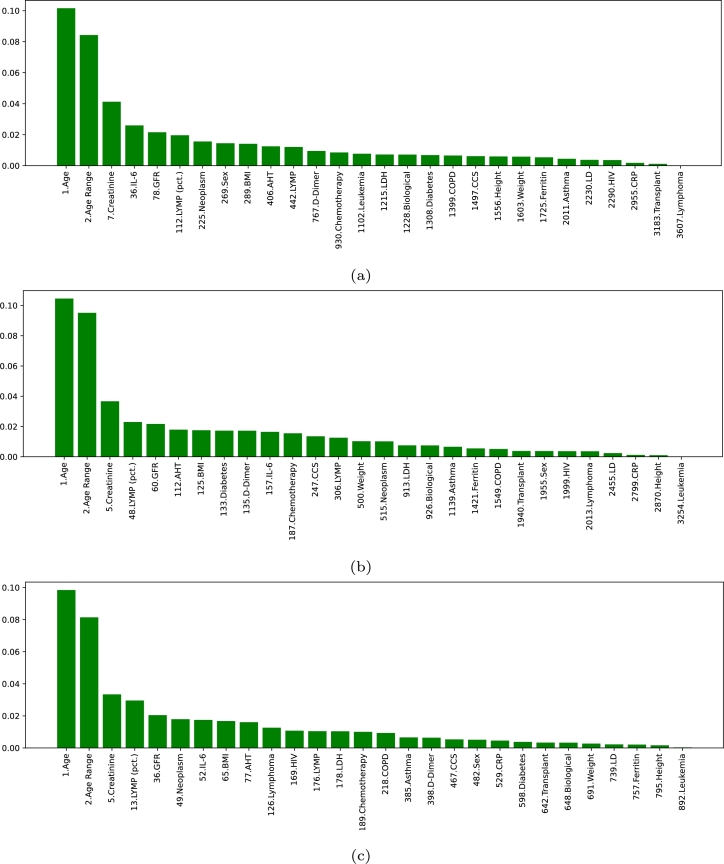
Ranking of the clinical features for the scenario II (Survival/Death), indicating their global position (*i.e.*, within the list composed of all the features), regarding the 3 different VGG-16 layers. (a) Clinical data + Imaging data (VGG-16 fc6). (b) Clinical data + Imaging data (VGG-16 fc7). (c) Clinical data + Imaging data (VGG-16 fc8).

Regarding the ranking of the clinical features in scenario II, that can be seen in Fig. 9, some remarkable conclusions can be extracted. The global appreciation is that these variables tend to be ranked higher in comparison with approach II. In fact, the variables Age, Age Range and Creatinine are always placed in the top 7 (being Age and Age Range always in positions 1 and 2, respectively, while Creatinine is placed in position 7 for VGG-16 fc6 and position 5 for fc7 and fc8). This is significant of the importance that clinical variables have in this scenario, an aspect that makes imaging features to lose relevance. From these discussions, it can be extracted that, contrary to the first scenario, the evidences that can be found in chest X-ray captures have a poorer correlation with the outcome of the patient, when estimating the risk of death. Consequently, the clinical variables are more decisive in this scenario. Nevertheless, another relevant conclusion that can be extracted is that, out of these 3 highly relevant variables, the rest of the clinical features are generally ranked lower than the top 30 for VGG-16 fc6 and VGG-16 fc7. Regarding the case of VGG-16 fc8, the additional features within the top 100 are LYMP (pct.) in position 13, GFR in position 36, Neoplasm in position 49, IL-6 in position 52, BMI in position 65 and AHT in position 77.

### 5.3 Comparison with other state-of-the-art approaches

To perform a comparison with other state-of-the-art approaches, it is important to note that there exists a lack of public datasets, making it difficult to do a comparative in fair conditions. This problematic is caused by the heterogeneous criteria that is used in the different health care institutions, an aspect that prevents the unification of different data sources to form a general broad dataset. Nevertheless, the dataset used in this work faithfully represents the profile of individuals from Western Societies (specially those from elderly cohorts), with a significant prevalence of conditions like diabetes and hypertension, an aspect that makes the provided study very relevant. Despite all the mentioned issues, we present a comparison with previous related state-of-the-art approaches, that can be seen in Table 7. It is important to note that, for our proposal, we report the highest-performing case for each scenario (*i.e.*, the one with the biggest value of AUC-ROC). As can be seen, from the 2 scenarios proposed in this work, the state-of-the-art contributions usually focus more on solving the task of death risk estimation. Regarding the scenario I (Non-Hospitalized/Hospitalized), the previous works only use sources of clinical data and, therefore, none of them propose a multimodal data fusion. Particularly, our proposal outperforms [38] in terms of accuracy and recall, while the results are in line for AUC-ROC and precision. The most significant drop is shown in terms of specificity. However, it is worth to mention that the previous work uses a much smaller dataset, with only 536 patients in contrast with 2040 patients available to our proposal. In the comparison with [36], there is a significant improvement in terms of recall, with a drop of the specificity and a slight raise of AUC-ROC.

**Table 7:** Comparison of the performance obtained in this work with other related state-of-the-art approaches. It is important to note that the presented works were compared in heterogeneous conditions, given that they use different datasets. CF: indicates that the work used clinical features. IF: indicates that the work used imaging features. It is important to consider that, for the works that include both CF and IF, we only report the performance obtained with multimodal data fusion.

	# of patients	CF	IF	Accuracy	F1-Score	Precision	Recall	Specificity	AUC-ROC
Scenario I: Non-Hospitalization/Hospitalization									
[38]	536			82.00%	-	94.00%	80.00%	87.00%	0.9100
[36]	2067			-	-	-	85.75%	60.44%	0.8415
Ours	2040			80.82%	88.21%	93.71%	83.36%	65.13%	0.8452

Scenario II: Survival/Death									
[37]	100			-	-	-	90.00%	95.60%	0.9590
[38]	536			81.00%	-	60.00%	89.00%	79.00%	0.9100
[62]	2547			75.00%	40.00%	27.00%	79.00%	74.00%	0.8500
[16]	4120			64.32%	-	-	80.47%	60.87%	0.8540
[36]	1783			-	-	-	75.87%	69.54%	0.7839
[40]	1795			-	-	-	72.00%	78.00%	0.8200
Ours	1761			77.42%	57.85%	51.68%	66.05%	81.03%	0.8285

In the case of the scenario II (Survival/Death), it can be seen that the global performance is competitive with other works, being the highest in accuracy after [38] and obtaining an AUC-ROC in line with the state-of-the-art. Regarding AUC-ROC, it can be seen that [37] and [38] get a high performance with 0.9590 and 0.9100, respectively. Nevertheless, these are the 2 contributions with the smallest datasets (536 patients in [38] and 100 in [37]). The rest of cases, that have a dataset over 1761 patients (considering our proposal as well), present very similar patterns and the AUC-ROC values are in the same line, with [36] a significant step below (with a value of 0.7839). Similar patterns are reflected regarding the trade-off between the performance when classifying the positive cases and when classifying the negative cases. Emami et al. [16] presents a high recall of 80.47% that compromises the specificity, with a value of 60.87%; Wu et al. [62] presents a more balanced situation, but with a higher recall (79.00%) than specificity (74.00%). Nevertheless, the positive predictive capabilities are compromised, given that the precision drops to 27.00%, obtaining a final F1-Score of 40.00% (17.85% lower than our proposal). Finally, Morís et al. [36] presents this imbalance with a recall of 75.87% and a specificity of 69.54% and Raman et al. [40] with a recall of 72.00% and a specificity of 78.00%. Generally, it can be concluded that the important imbalance of the used datasets makes the models to focus more on one class than the other to get a better global performance. Once again, it is necessary to remark that the main contribution of this work is the exhaustive analysis of the studied characteristics and scenarios, rather than the proposal of a method to outperform other state-of-the-art approaches and that the comparative is made under unfair conditions.

An important aspect to analyze, that has been slightly discussed previously, is the relationship between the dataset size and the AUC-ROC values. It is remarkable that the results point out a mixed picture, where some methods with larger datasets exhibit higher AUC-ROC values, but without a consistent trend across all comparisons. These findings suggest that, while dataset size can be an important factor, there are additional elements that can determine the classification performance. Other elements, such as the type of classifier, the type of proposed feature selection and extraction processes, the optimization techniques, data splitting and some specific characteristics of the dataset may play a role in the final AUC-ROC values achieved by each method.

## 6 Conclusions

In this work, we proposed an AI-based methodology that utilizes multimodal data fusion to enhance the clinical decision-making process. This fully-automatic methodology efficiently estimates hospitalization risk and mortality in COVID-19 patients by merging 28 clinical variables with imaging data extracted from chest X-ray images. The results demonstrate that the AI model can be trained with a reduced amount of features to obtain a competitive performance, requiring less computational resources, a very relevant aspect for clinical settings, where these resources are usually notably limited. Another demonstration extracted from the results is that adding imaging features to the original clinical data can be helpful to improve the performance of the machine learning classifier in the presented scenarios of risk estimation. However, the importance of each subset of features is different on each scenario. In particular, to estimate the risk of hospitalization, the imaging features gain a great relevance, while making the clinical data less relevant. On the opposite side, in the second scenario (estimate the risk of death), the clinical data has a great importance to determine the outcome. In general, the presented methodology provides a way to reduce the dimensionality of the original problem, making it more suitable to be implemented in clinical settings given the low availability of advanced computational resources that exists in these environments. This is reflected in the development of an efficient system that merges 2 different data sources that can outperform the use of both data sources independently. In fact, these metrics are obtained when only using a subset of clinical features and a subset of the imaging features, achieving a greater performance than training with the whole amount of features. The application of this methodology in the clinical practice could be easily implemented by the means of an integration with the already-existing infrastructure. The trained model could be used to process the data that has been stored in the corresponding servers of the hospital infrastructure.

As future works, this study could be complemented with more clinical variables to generate new knowledge about COVID-19 or other pathological scenarios (as the methodology could be easily extrapolated). The experimentation already performed could be also complemented with the exploration of alternative deep network architectures for feature extraction, including deeper or shallower architectures, to understand the impact of network complexity on classification performance. Focusing exclusively on the multimodal data fusion step, we could explore other more sophisticated feature fusion techniques and even the replacement of the current pipeline with an end-to-end architecture tailored to receive both inputs (clinical data and imaging data) simultaneously. Finally, another field worth to explore is the application of explainability in the scope of the proposed methodology. This incorporation could help the clinicians to adopt the methodology in their daily clinical practice. In fact, the discussion of the technology adoption is also another point worth to explore in future research.

## Ethics and consent declarations

This study was reviewed and approved by the local ethics committee of the “Sistema Público de Saúde de Galicia” with the approval number: 2020-007. All participants (or their proxies/legal guardians) provided written informed consent to participate in the study. The data were conveniently anonymized before being released from the corresponding radiology service.

## CRediT authorship contribution statement

**Daniel I. Morís:** Methodology, Software, Validation, Visualization, Writing – original draft. **Joaquim de Moura:** Methodology, Supervision, Validation, Writing – review & editing. **Pedro J. Marcos:** Data curation, Investigation, Methodology. **Enrique Míguez Rey:** Data curation, Investigation, Methodology. **Jorge Novo:** Conceptualization, Supervision, Validation, Writing – review & editing. **Marcos Ortega:** Conceptualization, Funding acquisition, Project administration, Supervision.

## Declaration of Competing Interest

The authors declare that they have no known competing financial interests or personal relationships that could have appeared to influence the work reported in this paper.

## Data Availability

The data and code used in this study are available online and have been referenced in the article.
